# Antarctic Fish as a Global Pollution Sensor: Metals Biomonitoring in a Twelve-Year Period

**DOI:** 10.3389/fmolb.2021.794946

**Published:** 2021-12-09

**Authors:** Alessandro Marrone, Daniele La Russa, Elvira Brunelli, Gianfranco Santovito, Mauro Francesco La Russa, Donatella Barca, Daniela Pellegrino

**Affiliations:** ^1^ Department of Biology, Ecology and Earth Sciences, University of Calabria, Rende, Italy; ^2^ Department of Pharmacy, Health and Nutritional Sciences, University of Calabria, Rende, Italy; ^3^ Department of Biology, University of Padova, Padova, Italy

**Keywords:** global pollution, antarctic fish, metals biomonitoring, metallothionein, bioaccumulation

## Abstract

Antarctica represents a unique natural laboratory for ecotoxicological studies as it is characterized by low internal pollutants emissions but high external contamination levels. Indeed, warm temperatures promote pollutant evaporation (low latitudes), while cool temperatures (high latitudes) promote its deposition from the atmosphere on land/water. Metals are the most important pollutants in ecosystems and represent a serious and global threat to aquatic and terrestrial organisms. Since 2000, the risks posed by metals have led many States to ratify protocols aimed at reducing their emissions. Endemic Antarctic organisms represent excellent bioindicators in order to evaluate the efficacy of global measures adopted to mitigate pollutants release into the environment. In this study (supported by PNRA18-00133), we estimated the metals contamination levels and the metallothionein-1 expression in liver samples of two Antarctic fish species, the icefish *Chionodraco hamatus* and the red-blooded *Trematomus bernacchii*, collected in the same area during 2002 and 2014. The chosen area is located in the Ross Sea, a unique area as it is also isolated from the rest of the Southern Ocean. The analysis of contamination trends throughout this period showed, in both species, a significant increase over time of metals bioaccumulation and metallothionein-1 expression. In addition, our result clearly indicated that the detoxifying ability of the two organisms analyzed greatly differs, probably due to haemoglobin presence/absence. Our work represents an important early step to obtain valuable information in conservation strategies for both Antarctic and non-Antarctic ecosystems.

## Introduction

The Antarctic continent is the southernmost part of the planet, but geographic isolation does not protect it from the negative impact of human activities. Long-range atmospheric transport is the main means by which contaminants reach Antarctica, as the Antarctic Polar Front’s presence strongly decreases the pollutant marine transport. Due to the low internal pollutant emissions but high external contamination levels, Antarctica is an excellent sensor for global pollution trends. Monitoring such levels is needed to evaluate the efficacy of global measures adopted to mitigate pollutant release into the environment. In addition, an effective control strategy is required for a better understanding of how they are distributed globally.

Metals are natural constituents of the earth’s crust and, through natural phenomena, are continuously released into the biosphere. Numerous anthropogenic activities have drastically altered their geochemical cycles and biochemical balance on a global scale. In recent decades, metals have polluted the environment from the poles to the tropics and from the mountains to the depths of the oceans (Cimboláková et al., 2020). These substances accumulate in Arctic and Antarctic areas where they have been identified in significant amounts despite the pollution sources being very distant. Warm temperatures favor pollutant evaporation (tropical and subtropical land surface), while cool temperatures (high latitudes) favor its deposition from the atmosphere on land/water (Wania and Mackay, 1996; Noyes et al., 2009). Climate change and global warming could enhance the transport/deposit of contaminants in polar areas and may be particularly problematic for species living at the edge of their physiological tolerance range where acclimation capacity may be limited (Noyes et al., 2009). Despite the polar environment vulnerability, studies on these territories are scarce and dated, though it has clearly emerged that trace metal contamination in organisms of the Southern Ocean are high in comparison to those from other oceans (Bargagli, 2000, 2008; Sanchez-Hernandez, 2000; Beltcheva et al., 2011; Roche et al., 2019).

In the present paper, we will focus on the metal’s contamination trend in Antarctica over a long period of time (12 years) using as bioindicators two fish species, the icefish *Chionodraco hamatus* and the red-blooded *Trematomus bernacchii*, collected in the same area during 2002 and 2014. The fish species are widely used as a bioindicator organism of Antarctic ecosystems due to their ability to accumulate pollutants, ecological relevance and physiological adaptations (Santovito et al., 2012a; Bargelloni et al., 2019; Garofalo et al., 2019; Tolomeo et al., 2019; Ansaloni et al., 2021). The selected area is located in the Ross Sea, which since 2017 has become the largest marine protected area in the world. The Ross Sea is located in a deep bay in southern Antarctica and is the largest ecosystem on the Antarctic continent, containing unique features and a level of biodiversity superior to other polar areas. The main feature that makes this area so unique is its physical conformation. In fact, this area remains isolated from the rest of the Southern Ocean because most of its surface water remains within its area and mixes little with the external water. This isolation ensures that the fish and larvae present do not disperse and mix with other organisms. Thus, not only is Antarctica a unique place for its marine fauna, but the fauna present in the Ross Sea has its characteristics that are only found there and nowhere else (Cummings et al., 2021).

Fish are widely recognized as the most relevant model for pollution monitoring in aquatic ecosystems (Van der Oost et al., 2003). In particular, Antarctic organisms, due to their endemic and highly adaptation ability to extreme conditions, represent excellent bioindicators to analyze the trend of metal bioaccumulation over time. In fish, metals can enter the body through the gills or the intestinal wall and then distributed to the tissues (Tchounwou et al., 2012). Both essential and non-essential metals can exert toxic actions, whereby intracellular metals levels are highly regulated by metal-binding proteins such as metallothioneins (Wang et al., 2014; Kan et al., 2019). Metallothioneins (MTs), a family of highly conserved cysteine-rich proteins evolved in all eukaryotes from unicellular to vertebrate (Formigari et al., 2010; Santovito et al., 2012b, 2015, 2021), are involved in several cellular processes and play a key role in cellular protection from actions of harmful agents such as metal and reactive oxygen species (Santovito et al., 2008; Ruttkay-Nedecky et al., 2013; Wang et al., 2014; Kan et al., 2019).

We choose the liver as a target organ since it is implicated in the regulation of metabolic pathways, homeostasis, and detoxification and it may represent a good indicator of bioaccumulation and detoxification after metal exposure (Qu et al., 2014; Macirella et al., 2016, 2019; Macirella and Brunelli, 2017). The objective of the current research was, on the one hand, to monitor the fragile Antarctic ecosystem and, on the other hand, to obtain critical information on the global trend of metal pollution to drive the conservation strategies for the Antarctic and non-Antarctic ecosystems.

## Materials and Methods

### Animals

Adult specimens of two species of benthonic fish, the red-blooded *Trematomus bernacchii* Boulanger (Notothenidae) and the icefish *Chionodraco hamatus* Lönnberg (Channichthydae), were caught using nets at depths of 80 and 150 m during the XVII (2001–2002) and XXIX (2013–2014) Italian Antarctic expeditions in the Terra Nova Bay, Ross Sea, Antarctica (74° 41′ 42″ S, 164° 07′23″ W). Target fish are endemic and non-migratory species, and identification was based on descriptions of Fisher and Hureau (1985). The sampling, performed in a 12-years range, was operated so as to ensure a broad geographical representation and adequate homogeneity, according to the highest standards in the use of these organisms for biomonitoring activities. We used 5 adult specimens of both sexes for each species: -2002- *T. bernacchii* 294.2 ± 58.08 g, 28.6 ± 5.36 cm; *C. hamatus* 396 ± 37.81 g, 36.1 ± 2.31 cm; -2014- *T. bernacchii* 298.6 ± 53.89 g, 28.2 ± 5.71 cm; *C. hamatus* 389.4 ± 29.94 g, 35.62 ± 2.07 cm. After capture and relaying, each animal was anesthetized with ethyl 3-aminobenzoate methanesulfonate salt (0.065 g/L; Sigma; St. Louis, MO, United States) and then weighed. Samples of both liver (2002 and 2014) and plasma (2014) properly stored (frozen in liquid nitrogen and stored at −80°C) were obtained from previous Antarctic expeditions and used for *ex novo* analysis. Correctly stored plasma samples from 2002 could not be found.

### Biomonitoring of Pollutants

We estimated the contamination levels of 10 metals: strontium (Sr), chromium (Cr), cobalt (Co), nickel (Ni), zinc (Zn), arsenic (As), selenium (Se), molybdenum (Mo), cadmium (Cd), and lead (Pb). The liver content of each metal was determined by a quadrupole Inductively Coupled Plasma-Mass Spectrometer (ICP-MS, PerkinElmer, model Elan DRCe), by means of solution nebulization. Before analysis, samples were mineralized by acid digestion using a microwave oven. The liver samples were dried under a laminar flow hood for 24 h and weighted into digestion vessels. After adding 10 ml of ultrapure HNO_3_, digestion vessels were capped and placed into a 12-position turntable CEM (Mars 5) microwave oven. A ramped temperature control program was applied: ramp to 180°C for15 min, followed by 15 min cooling down. The temperature was monitored in a control vessel by an armored fiber-optic temperature control probe, and the pressure was monitored by an internal pressure control system. After cooling down, microwave vessels were vented, and extractions were diluted to a final volume of 100 ml using Milli-Q water. Finally, the solution is preserved at a temperature of 4°C. In each analytical sequence, procedural blanks and reference materials were included. The precision and accuracy of the applied analytical method were estimated on Certified Reference Material (CRM), Tort 2 (lobster hepatopancreas).

### Western Blotting Analysis

Samples of cell-free liver extract containing 50 μg of proteins were heated for 5 min in Laemmli buffer (Sigma, St Louis, MO, United States), separated by SDS PAGE using 15% gel in a Mini *trans*-blot transfer cell, and then transferred to nitrocellulose membrane using a mini *trans*-blot (Bio-Rad Laboratories, Hercules, CA, United States). The membrane was blocked with TBS-T buffer containing 5% non-fat dry milk. For immunodetection, the blots were incubated overnight at 4°C with monoclonal mouse anti-MT antibody (Enzo Life Sciences) diluted in TBS-T buffer containing 5% BSA. The blots were then washed and incubated with a peroxidase linked secondary antibody (Santa Cruz). Protein loading was verified using anti-β-actin antibody (Santa Cruz). Immunodetection was performed by using an enhanced chemiluminescence kit, and autoradiographs were obtained by exposure to X-ray Films. Immunoblots were digitalized, and the densitometric analysis of the bands obtained was carried out using WCIF ImageJ based on 256 grey values (0 = white; 256 = black). Quantification of the bands was obtained by measuring (5 times on each band) the mean optical density of a square area after the background area was subtracted. The results of absorbance measurements and the grey values obtained from the densitometric analysis were expressed as mean ± SE of five determinations for each sample.

### Measurement of Plasma Oxidative Status

Plasma oxidative status was determined by using photometric measurement kits and a free radical analyzer system with a spectrophotometric device reader (FREE Carpe Diem, Diacron International, Grosseto, Italy), which are routinely used in our laboratory in both human and animal models (Brunelli et al., 2017; La Russa et al., 2017; La Russa et al., 2019; Russa et al., 2019; La Russa et al., 2020; La Russa et al., 2021; Perri et al., 2021). Plasma oxidative stress was assayed using a Diacron-reactive oxygen metabolite (dROM) test. Results are expressed in Carratelli Units (UC; 1 UC = 0.8 mg/L of hydrogen peroxide). Total plasma antioxidant capacity was assayed using a biological antioxidant capacity (BAP) test. Results are expressed in µmol/L of the reduced ferric ions.

### Statistical Analysis

Data were analyzed using the GraphPad/Prism version 5.01 statistical software (SAS Institute, Abacus Concept, Inc., Berkeley, CA, United States). Statistical differences were examined using two-way Anova followed by Bonferroni’s multiple comparison tests, and the significance level was considered at *p* < 0.05. Results are expressed as the mean ± standard error (SE).

## Results

### Metals Bioaccumulation

**FIGURE 1:**
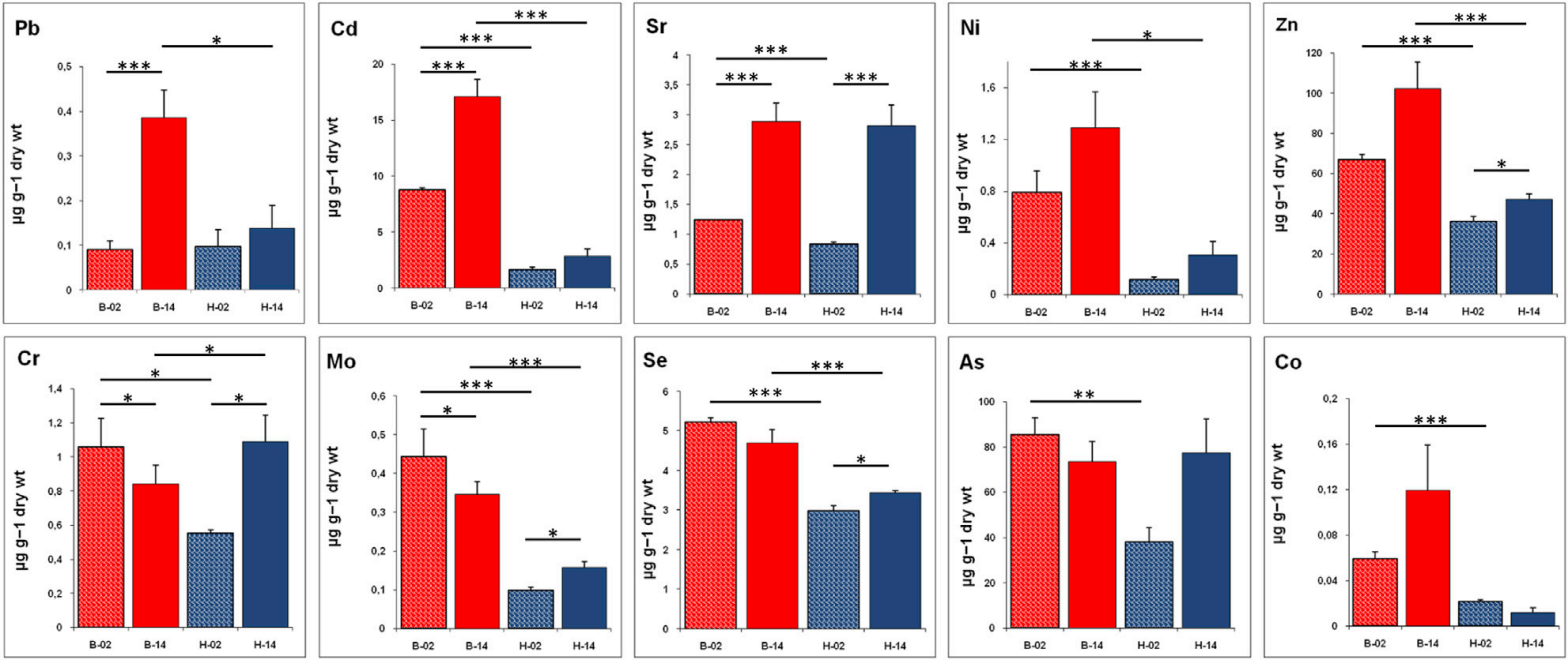
Bioaccumulation level of metals (Pb, Cd, Sr, Ni, Zn, Cr, Mo, Se, As, and Co) in liver of *T. bernacchii* (B) and *C. hamatus* (H) in 2002 (02) and 2014 (14). Data are expressed as means ± SE (n = 5); statistical differences were evaluated by two-way Anova followed by Bonferroni’s multiple comparison tests (*p < 0.05; **p < 0.025; ***p < 0.005).

In the considered period, in the liver of *T. bernacchii*, a significant increase has been detected for Cd, Sr, and Pb (Figure 1). It is also interesting to underline the increase in Zn, Ni, and Co concentrations, although the differences are not statistically significant. Moreover, in this species, a significant decrease in liver concentration has been detected for Mo and Cr, whereas Se and As decrease insignificantly. In *C. hamatus* we recorded an increase, from 2002 to 2014, of all tested metals in the liver tissue except for Co, which shows a slight decrease from the low initial value (Figure 1). Interestingly, in this hemoglobinless organism, the bioaccumulation levels are much lower than in the red-blooded species *T. bernacchii*, especially in the first sampling carried out in 2002. The increase in hepatic concentrations is significant for Sr, Se, Mo, Cr, and Zn. A great increase in Sr, Cr, and As contents is particularly evident as the initial value is doubled, reaching levels comparable to the values found in *T. bernacchii*.

### Metallothionein Expression

**FIGURE 2:**
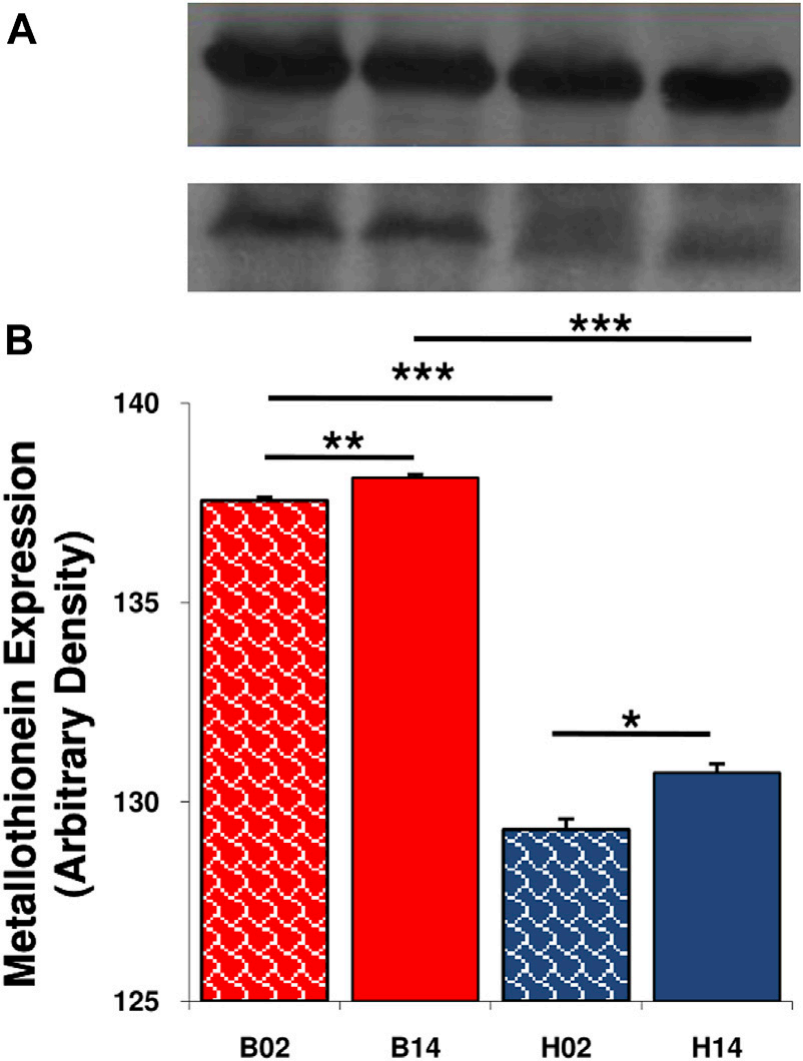
Expression level of metallothionein in liver of *T. bernacchii* and *C. hamatus* in 2002 (02) and 2014 (14). **(A)** Representative immunoblots of metallothionein and β-actin. **(B)** Densitometric analysis of protein levels; data are means ± SEM of seven determinations for each animal (n = 5); statistical differences were evaluated by two-way Anova followed by Bonferroni’s multiple comparison tests (*p < 0.05; **p < 0.025; ***p < 0.005).

Comparing samples collected in 2002 and 2014, we observed a significant increase in metallothionein-1 expression in both species (Figure 2). The levels were always higher in the red-blooded species *T. bernacchii* than in the icefish *C. hamatus*.

### Plasmatic Oxidative Status

**FIGURE 3:**
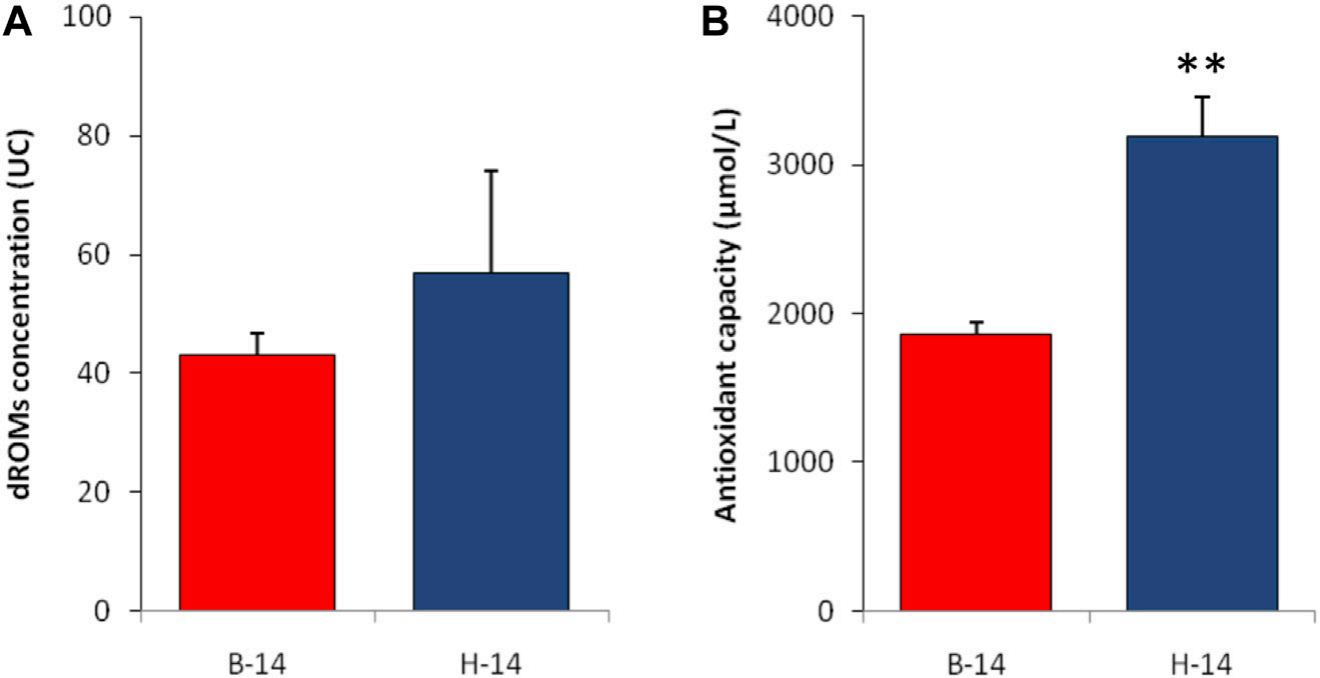
Plasmatic values of reactive oxygen metabolite **(A)** and biological antioxidant capacity **(B)** test in liver of *T. bernacchii* (B) and *C. hamatus* (H) in 2014 (14). Data are expressed as means ± SE (n = 5); statistical differences were evaluated by two-way Anova followed by Bonferroni’s multiple comparison tests (**p < 0.025).

The analysis of the plasmatic oxidative balance showed some differences between the two species. The icefish showed higher levels of hydroperoxides, although this difference is not statistically significant. A statistically significant difference was found in the levels of antioxidant capacity, once again in favor of *C. hamatus* (Figure 3).

## Discussion

This research aims to employ Antarctica as a global pollution sensor by using its endemic organisms as bio-indicators. Despite their remoteness, the polar regions are increasingly subject to anthropogenic contamination. The high degree of endemism and the unique eco-physiological adaptations make Antarctic species particularly sensitive to contaminants (Bargagli, 2008; Garofalo et al., 2009). The measure of metals that reach isolated and putatively uncontaminated areas as the polar zones allows us to evaluate the risk of such substances that travel across the world and accumulate in ecosystems. In the last few years, several studies have concerned the metal contamination in various organic and inorganic matrices of the Antarctic continent (Koppel et al., 2019; Potapowicz et al., 2019; Pilcher et al., 2020; Webb et al., 2020; Xu et al., 2020). Although the structure and dynamics of Antarctic ecosystems make them valuable tools for assessing past and current global pollutant levels, almost no (contamination) data before 2000 is available, and very few are available for the years 2000–2015 (Bargagli, 2000, 2008; Twining and Baines, 2013; Vodopivez et al., 2015).

To fill this gap, we have chosen to analyze the metal contamination trends in 12 years (in 2002 and 2014) and evaluate both bioaccumulation and some physiological responses, such as metal homeostasis and antioxidant defenses of model organisms. The use of organisms as a biological indicator to determine trace metal pollution is particularly useful because organisms concentrate metals from the environment, but they may also represent a moving time-averaged value for the metal biological availability.

The first outcome that appears noticeable by an overall analysis of our results is that the two fish, although both are benthic species (Eastman, 1993), show different levels of metal bioaccumulation, specifically the red blooded species *T. bernacchi* exhibit higher levels with respect to the icefish *C. hamatus,* and this is particularly evident in the 2002 sampling. It is known that the tissue levels of different metals vary as a result of different uptake pathways, and metals concentration exhibited a large variability in the same tissue according to the species (Terra et al., 2008; Bervoets et al., 2013; El-Moselhy et al., 2014; Mahboob et al., 2016). We can suppose that the ecological and behavioral features of species might play a role in the processes of uptake and accumulation of the metals by organisms, and also local environmental might influence the different patterns observed in the two considered species. Moreover, we must consider that the two species analyzed show profound physiological differences due to the presence/absence of hemoglobin and the different redox balance levels showed.

In the environment, organisms are simultaneously exposed to multiple metals and their absorption can vary due to additive, antagonistic or synergistic phenomena (Tchounwou et al., 2012). Indeed, essential metals are important constituents of both oxidative stress-related enzymes and metalloenzymes involved in hemoglobin formation (Tchounwou et al., 2012). Our results showed a significant increase over time in the bioaccumulation of many metals and, in particular, Pb, Cd, Sr, Ni, and Zn showed a remarkable growth trend in bioaccumulation in both species analyzed. In small amounts, Pb occurs naturally in the environment, however, anthropogenic activities contribute to the high release of this dangerous toxicant. In particular, the fossil fuel burning and the production of leadacid batteries and paint represented the main source of lead pollution. Human exposure to lead represents a serious health problem as it is the most systemic toxicant that affects several organs in the body including the kidneys, liver, central nervous system, hematopoietic system, endocrine system, and reproductive system (Flora et al., 2012; Wani et al., 2015). In recent decades, many States ratified protocols aimed at decreasing emissions and the lead release has been significantly reduced at the industrial level, although the most marked effect was achieved with the elimination of Pb in gasoline. In this research, we have focused on a very important period of time to evaluate the environmental effect of the ban on lead from gasoline as 2002 is the year in which all states have effectively adhered to this protocol. Despite the effective and significant reduction of emissions, our results clearly demonstrate that lead bioaccumulation has considerably grown in both species analyzed, highlighting that global pollution mitigation measures take extremely long time. However, we must not forget that this increase in Pb bioaccumulation detected in our Antarctic organisms could also derive from remobilization of both natural and industrial Pb due to increased surface melt duration and extent caused by increased temperature over the last years (Ndungu et al., 2016). In addition to Pb, also the other non-essential metals Cd, and Sr showed significant bioaccumulation in both fish species analyzed. In particular, the Sr levels detected in 2014 are significantly higher than in 2002 also in the icefish, which, compared to the red-blooded species, generally shows lower levels of bioaccumulation. Concerning the Cd levels, it is important to mention that this metal is characterized by a high accumulation coefficient and high chemical similarity to Zn, thus Cd easily enters various enzymatic reactions instead of Zn altering biochemical processes (Cimboláková et al., 2020). In fish, Pb, Cd, and Sr pollution are particularly serious as their assimilation mainly occurs through the chloride cells of gill epithelium, since all three of these non-essential metals present high affinity with calcium uptake mechanisms (Olsson et al., 1998; Beltcheva et al., 2011).

The analysis of Ni and Zn levels showed a consistent increase from 2002 to 2014 in our bio-indicator organisms. Ni and Zn are essential micro-nutrients that are required for various biochemical and physiological functions (Tchounwou et al., 2012). Specifically, Zn is a key factor in a variety of biological processes, as a structural, catalytic, and intracellular and intercellular signalling component (Frassinetti et al., 2006), while Ni, although its biological function is still somewhat unclear, is involved in the maintenance of metabolic homeostasis (Kumar and Trivedi, 2016). Although the essential functions of these metals, their accumulation can cause structural lesions and functional disturbances as an excessive absorption exceeds the detoxifying ability of the organism (Javed and Usmani, 2019). The other essential micro-nutrients analyzed (Cr, Mo, Se, and Co) showed different bioaccumulation patterns in the two Antarctic fish evaluated in this study: Cr, Mo, and Se decrease significantly from 2002 to 2014 in the red-blooded teleost *T. bernacchii* while in the icefish all three increase significantly and in the case of Cr reaching values even higher than those of the red-blooded species. The increase of many tested elements in hepatic tissues of both *C. hamatus* and *T. bernacchii* supports the hypothesis of an increase in environmental concentrations of metal ions from 2002 to 2014.

MTs are proteins able to bind metals and represent an excellent biomarker in the assessment of environmental pollution of these xenobiotics (Brunelli et al., 2011). The induction of metallothionein biosynthesis in fish after metal exposure is well known (Bargelloni et al., 1999; Sinaie et al., 2010; Wang et al., 2014). Analyzing samples from 2002 to 2014, we observed a significant increase in metallothionein expression in both species. It is also evident that the levels are always higher in *T. bernacchii* than *C. hamatus* leading us to suppose a species-specific response to the presence of metals in seawater. This result could be also in relation of the Cd accumulation as it is well known that this metal is one of main metallothionein inducers (Price-Haughey et al., 1987; Kovarova et al., 2009; Le Croizier et al., 2018). However, we must not forget that MTs, in addition to their role of homeostasis/detoxification from metals, represent a significant element in maintaining the redox balance of the organism (Sevcikova et al., 2011; Wang et al., 2014; Ma’rifah et al., 2019).

The Antarctic environment, characterized by low seawater temperature and extreme seasonality of light and food supply, makes its organisms more vulnerable compared to temperate species to harmful agents such as metal and reactive oxygen species (ROS). The ROS scavenging function of MTs in Antarctic fish could be very important in relation to the high oxygen partial pressure of their environment. These animals live in cold waters where the dissolved oxygen concentration is very high as a consequence of low temperature that increases gas solubility. Thus, it is possible that the MT system should be involved in the ROS buffering, and in particular in the red-blooded species *T. bernacchii*, which exhibits high levels of MT expression, and consequently had an optimal redox balance, with d-ROM values lower than other seawater fish such as *Pagrus major* (Hossain et al., 2016). Also the icefish have a low plasmatic level of oxidative stress index despite low values of MT expression. It is probable that in this species the greatest physiological role against the risk of oxidative stress is played by antioxidant enzymes. The enzymatic component of the antioxidant defense system is particularly important in Antarctic fish, which have evolved under selective pressure represented by the constant production of high levels of ROS. Recent studies have shown that the antioxidant enzymes of these fish possess molecular adaptations affecting both their gene structure and the regulation of expression (Sattin et al., 2015; Tolomeo et al., 2016, 2019; Nicorelli et al., 2018; Chatzidimitriou et al., 2020).

In conclusion, the present work provides new information on the distribution over time of metal-ions in fish from Antarctica, and is an important contribution to study the responses of these organisms against environmental pollution. In addition, our results provide a general scenario of worldwide pollution, thus supporting the conservation strategies for Antarctic and non-Antarctic habitat protection.

## Data Availability

The raw data supporting the conclusion of this article will be made available by the authors, without undue reservation.
